# Navigating Suicide-Related Online Content in Individuals With Lived Experience of Suicidality: Cross-Sectional Survey

**DOI:** 10.2196/92693

**Published:** 2026-10-20

**Authors:** Jacob Greaves, Dafni Katsampa, Lydia Grace, Ciara Harris, Anna Lavis, Ian Marsh, Lisa Marzano

**Affiliations:** 1 Middlesex University, London, England, United Kingdom; 2 Samaritans, London, United Kingdom; 3 University of Birmingham, Edgbaston, Birmingham, England, B15 2TT, United Kingdom; 4 Warwick Medical School, University of Warwick, Coventry, United Kingdom; 5 Canterbury Christ Church University, Canterbury, United Kingdom

**Keywords:** social media, suicide content, lived experience, user intent, online safety

## Abstract

**Background:**

Engagement with suicide-related content online is often assumed to be harmful, yet many individuals with lived experience describe such content as supportive or even life-preserving. Despite policy and clinical concerns, little is known about how people intentionally or unintentionally encounter this content, how they interpret it, and how these experiences shape perceived harm or benefit.

**Objective:**

This study aims to explore how intent and context influence the ways in which individuals with lived experience of suicidality encounter, interpret, and respond to suicide-related online content, and to identify factors associated with perceived harm and benefit.

**Methods:**

An exploratory cross-sectional survey was conducted with a convenience sample of 713 UK-based participants aged 16 to 60 years and older, 95.9% (n=684) of whom reported having lived experience of suicidality. Quantitative data were analyzed using descriptive and inferential statistics, including chi-square tests, to examine associations between engagement patterns, intent, and suicidality. Free-text responses were coded using content analysis to identify categories relating to motivations for engagement, emotional responses, and perceived impact.

**Results:**

Participants reported a broad range of experiences. While 30.0% (214/713) described suicide-related content as contributing to suicidal thoughts or attempts, others reported relief, validation, or reduced feelings of isolation, with 21.0% (150/713) attributing content to preventing an attempt. Clear differences emerged between participants who actively sought out content (“seekers”) and those who encountered it unintentionally (“stumblers”). Seekers more frequently reported positive or understanding responses, often in the context of unmet offline support needs, whereas stumblers were more likely to report shock or distress. Approximately 39.9% (151/378) of participants described their engagement as neither intentional nor accidental, potentially reflecting a blurred boundary between passive exposure and active seeking, as well as the difficulty individuals face in accurately identifying and articulating their own motivations in relation to suicidality.

**Conclusions:**

Engagement with suicide-related online content cannot be characterized as uniformly harmful. Outcomes depend on user intent, emotional state, and the context of engagement, and they may change over time. Clinical, regulatory, and policy approaches should move beyond risk-only frameworks to account for the nuanced and sometimes supportive roles such content plays in how individuals make sense of suicidal thoughts, while still protecting those at risk.

## Introduction

Unlike most European countries, the United Kingdom has seen rising suicide rates in recent decades, since around 2005 [1]. While explanations remain contested, evidence suggests a complex interplay of factors, including increased prevalence of mental health disorders and broader socioeconomic stressors [1,2]. Another prevalent explanation notes the contemporaneous proliferation of social and other online media that may present real harms to users’ mental health [3,4].

In response, regulatory bodies in the United Kingdom have tended to prioritize protective interventions. The UK government’s Online Safety Act (OSA), for example, reflects a precautionary approach that emphasizes reducing exposure to potentially harmful material, often through content moderation and removal strategies [5]. While such policies aim to safeguard online users, they may also carry unintended consequences, such as limiting access to content that some individuals find beneficial [6]. Existing regulatory frameworks may not yet fully account for differences in user intent or the nuanced ways in which suicide-related content is encountered and received [7-9].

Suicide-related content online has, therefore, emerged as both a site of public health concern and a potential psychosocial intervention. On the one hand, engaging with suicidal content is associated with an increased risk of suicidal behavior [10]. One explanation is that suicide-related posts can foster “contagion,” especially among adolescents, through assortative relating [11] or other reinforcement of suicidal behaviors [10]. On the other hand, online environments can be a space where people share experiences that are otherwise silenced, helping them feel seen and understood. Such content on social media may create a much-needed sense of connection and support, especially for those who lack meaningful offline relationships and a sense of belonging [9].

Despite the bidirectional effects, research has predominantly focused on associations between encounters with online suicide content and harm, often neglecting the meanings that individuals ascribe to this content and the psychosocial drivers underlying their engagement. While some differentiation has been made between individuals who encounter such content incidentally and those who actively seek it [8], the ways in which the intent underlying these encounters might intersect with users’ affective states, engagement patterns, and perceived impact remain poorly understood.

The present study addresses this gap through a mixed methods survey of individuals with personal experience of suicidal thoughts and/or behaviors. We explored people’s encounters with suicide-related content online, their motivations and modes of engagement, and how participants’ reflected on differences between their initial and more recent encounters with suicide-related content. In doing so, we move beyond a nonagentic exposure-as-risk framing to additionally consider how individuals’ intent, emotional state, and content type interact in shaping outcomes.

## Methods

### Study Design

This study draws on data from a UK-based online survey conducted between June 2021 and January 2022. The survey used a cross-sectional design, combining multiple choice and open-ended questions to explore participants’ experiences of discovering and engaging with suicidal and self-harm–related content online.

### Ethical Considerations

Informed consent was obtained from all participants prior to their involvement in the study. Participants were provided with an information sheet outlining the purpose of the research, procedures, potential risks and benefits, data handling, and their right to withdraw at any time without consequence. The information sheet also detailed plans to publish nonidentifiable data. Consent was indicated by participants ticking a box to confirm their voluntary agreement to participate. Where applicable, this research adhered to the ethical principles outlined in the World Medical Association Declaration of Helsinki. The research received ethical approval from the Middlesex University Psychology Department’s Research Ethics Committee (reference number 17869). Participation was anonymous.

### Recruitment and Materials

As a precaution for participants’ well-being, individuals experiencing current suicidal or self-harming thoughts or who had attempted suicide within the past month were advised against participating in the study. Recruitment was conducted via a range of channels to ensure a broad and diverse sample, including social media platforms (eg, Samaritans’ Twitter or X feed), newsletters (eg, National Suicide Prevention Alliance), online forums (eg, PistonHeads), and specialist panels and insight groups (eg, Autistica).

The survey collected information about participants’ history of suicidal and self-harm thoughts and behaviors. Sociodemographic information was collected to understand the sample composition (Multimedia Appendix 1). Subsequent sections explored experiences of encountering and engaging with suicide and self-harm–related content across various online platforms, along with the perceived emotional and behavioral impacts of this content.

Participants were routed only to questions relevant to them. Expected variation in denominators reflects a combination of the survey’s branching design and item nonresponse. Participants who did not meet the eligibility criteria for a branched item were not asked that question and were not counted as missing. For example, those who had not encountered suicide-related content online did not answer further questions about that content. Questions about the content were designed to quantify and contextualize associations between different types of content or engagement and outcomes such as emotional well-being, distress, self-harm, and suicidality both immediate and longer-term.

Additional questions focused on patterns of online behavior and the use of support services, including third sector and National Health Services (NHSs). These data were used to identify when encountering this content is most likely to be harmful and for whom. Participants also provided perspectives on content moderation and blocking mechanisms, as well as suggestions for improving online safety and support. In this paper, we report participants’ experiences of online content relating specifically to suicide; responses relating to self-harm content and perspectives on content moderation and other online support mechanisms are reported separately.

### Data Analyses

Quantitative data were analyzed in SPSS (version 29.0; IBM). Descriptive statistics were used to summarize participant characteristics and the prevalence of posting and responding to online suicide content.

Free-text responses were coded using content analysis [12]. An initial coding framework was developed and refined through an iterative process. Approximately 10% of responses were independently coded by 2 researchers (LK and CH) to identify ambiguities, compare interpretations, and refine the coding scheme. Regular consensus meetings involving LK, CH, and LM were held throughout the analysis to discuss discrepancies and agree on code definitions and their application (see Multimedia Appendix 2 for the content analysis codebook). Formal interrater reliability statistics were not calculated because the purpose of independent coding was to refine and calibrate the coding framework through discussion and consensus, rather than to assess coder agreement as an outcome.

Content categories were then used to generate descriptive statistics, using frequencies and/or percentages, as appropriate, with denominators varying in relation to individual variables because of missing information. Inferential statistics, including chi-square tests, were then used to assess statistically significant differences and associations, eg, regarding the impact of online suicide content or content removal and/or restrictions on different groups. For instance, to explore differences in relation to key dimensions of suicidality as identified in previous literature [13], we examined patterns of exposure and engagement with online content between “ideators” (those who have experienced suicidal thoughts but never attempted suicide) and “enactors” (those who have made a suicide attempt).

Structured and free-text responses relating to reactions to the first encounter with suicidal content were categorized based on emotional valence. The initial content coding was conducted independently by 2 coders. The higher-order valence categories and classification rules were developed through consensus discussion within the research team. The final valence classification was then derived in SPSS from the agreed first-order codes using these predefined rules.

Categories were defined as follows: “negative” (eg, unsettled, scared, shocked, sad, and angry), “positive” (eg, resilient, comforted, less alone, and sense of belonging), and “curious” where no clear valence could be drawn but where exploratory intent was a feature (which included statements similar to “wanting to try to know or see more”; “wanting to try out what I saw”; and excludes “wanting to talk to someone about what I saw”). During coding, a distinct subcategory within “positive” responses emerged from free-text responses: “positive in relation to others” (eg, wanting to help), which captured altruistic motivations and concern for others' well-being.

The higher-level groupings were as follows: negative (including negative and curious), positive (including positive and curious), mixed (defined as a mix of positive and negative reactions), and curious-only (in which only curiosity-related reactions were given). The decision rule for groups is as follows: when curiosity cooccurred with positive and/or negative responses, it was treated as an accompanying feature rather than determining group membership. Participants were assigned to the positive, negative, or mixed group according to the valence of their noncuriosity responses. Curiosity determined group membership only when it was the sole type of response reported. For higher-level groupings, participants were classified according to the overall pattern of reactions reported.

Inferential analyses were conducted using chi-square tests to assess associations among categorical variables. Mann-Whitney *U* tests were used for ordinal or nonnormally distributed variables, including comparisons of content-type exposure between seekers and stumblers. No formal correction for multiple comparisons was applied given the exploratory nature of the study; findings were interpreted with appropriate caution throughout. Given the exploratory nature of the study, no formal correction for multiple comparisons was applied, as doing so would risk inflating type II error in a study primarily intended to generate hypotheses rather than test them. However, we acknowledge this as a limitation and recommend that findings from inferential tests be interpreted with caution and treated as indicative rather than confirmatory, pending replication in hypothesis-driven studies.

Missing data were not imputed. Due to the survey’s branching design, not all participants were routed to every question; analyses were therefore conducted on available data for each variable, with denominators reported throughout to ensure transparency.

Quotes are presented in the *Results* section alongside age and gender to give context to the data.

## Results

### Participant Characteristics

A total of 713 participants responded to the survey, forming a convenience sample of UK residents aged 16 to 60 years and older and are summarized in Table 1. Participants were asked about basic demographic descriptions of age, gender, and ethnicity. The age range of participants was 16 to 24 years (n=219, 30.7%), 25 to 39 years (n=234, 32.8%), and more than 40 years (n=253, 35.5%). Gender was split between woman or girl (n=435, 61.0%), man or boy (n=185, 25.9%), and transgender or other (n=86, 12.1%). Participants described their ethnicity as White (n=608, 85.3%), Asian or Asian British (n=35, 4.9%), mixed ethnicity (n=26, 3.6%), Black or Black British (n=21, 2.9%), and other (n=15, 2.1%).

Lived experience of suicide was prevalent in the sample, with 85.1% (588/691) having experienced suicidal thoughts, a high proportion of which occurred within the last 6 months (321/588, 54.6%). The proportion of the sample who had made attempts was 42.8% (287/670), of which 21.3% (61/287) occurred within the last 6 months. More broadly, 95.9% (684/713) of the sample reported some personal or family experience of self-harm or suicidality.

Participants’ online lives were assessed through questions about the time they spent online and which websites they visited regularly. Out of 710 participants, 94.5% (n=671) of the sample spent at least an hour online every day, and around half (n=365, 51.4%) spent at least 4 hours online per day. The most regularly visited sites by participants were YouTube (469/731, 65.8%), Facebook (439/713, 61.6%), Instagram (384/713, 53.9%), Twitter (328/713, 46.0%), and TikTok (199/713, 27.9%).

**Table 1.** Sample characteristics (N=713)^a^.

Characteristic	Values
Age group (y), n (%)	
16‐24	219 (30.7)
25‐39	234 (32.8)
>40	253 (35.5)
Gender, n (%)	
Woman or girl	435 (61.0)
Man or boy	185 (25.9)
Transgender or other	86 (12.1)
Ethnicity, n (%)	
White	608 (85.3)
Asian or Asian British	35 (4.9)
Mixed ethnicity	26 (3.6)
Black or Black British	21 (2.9)
Other	15 (2.1)
Lived experience of suicidality, n/N (%)	
Any personal or family experience of suicidality or self-harm	684/713 (95.9)
Ever experienced suicidal thoughts	588/691 (85.1)
Of whom: in the last 6 months	321/588 (54.6)
Ever made a suicide attempt	287/670 (42.8)
Of whom: in the last 6 months	61/287 (21.3)
Suicidality type, n/N (%)	
Ideators (suicidal thoughts, no attempt)	280/567 (49.4)
Enactors (history of suicide attempt)	287/567 (50.6)
Online behavior, n/N (%)	
At least 1 hour online per day	671/710 (94.5)
At least 4 hours online per day	365/710 (51.4)
YouTube (regular use)	469/713 (65.8)
Facebook (regular use)	439/713 (61.6)
Instagram (regular use)	384/713 (53.9)
Twitter or X (regular use)	328/713 (46.0)
TikTok (regular use)	199/713 (27.9)
Exposure to suicide-related online content, n/N (%)	
Ever seen suicide-related content online	455/616 (73.9)
Seen it 5 or more times	263/616 (42.7)
First encountered before age 18 years	173/359 (48.2)
First encountered before age 14 years	122/359 (33.9)
Mode of first encounter with suicide-related content, n/N (%)	
Stumbler (unintentional)	151/378 (39.9)
Seeker (intentional)	76/378 (20.1)
Neither or ambiguous	151/378 (39.9)

a Denominators vary across variables due to missing data and the survey’s branching design. Percentages are rounded to one decimal point. The ideator or enactor classification is based on self-reported history of suicidal thoughts and/or attempts.

### Experiences of Online Suicide Content

Three quarters of participants (455/616, 73.9%) reported having seen suicide-related content online at least once, with 42.7% (263/616) having seen it 5 times or more. Finding such content online was significantly associated with lived experience of suicidality (*χ*²_1_=12.28; n=608; *P*<.001). Among those with a history of suicidal thoughts or behaviors, 77.2% (392/508) had seen suicide-related content, compared with 59.6% (53/89) of those without such experiences.

Encountering suicidal content was highly prevalent even among the youngest participants (aged 16‐25 y), with 79.2% (156/197) reporting that they had seen such content online. Remarkably, across the wider sample, 48.2 % (173/359) said they first encountered it before they were 18 years of age, and a third by 14 years of age (122/359, 33.9%).

### Intent of Stumbling vs Seeking

Participants who reported encountering suicide-related content were asked whether this occurred accidentally, intentionally, or in a way that did not fit either category. Among the 378 participants, as outlined earlier, the analysis grouped participants into 2 broad categories based on the nature of their first encounter: “stumblers,” who encountered the content unintentionally (n=151, 39.9%), and “seekers,” who actively sought it out (n=76, 20.1%). Not all participants answered subsequent item-level questions about content type; therefore, sample sizes vary across analyses. Notably, 39.9% (n=151) of participants reported that finding content for the first time was neither accidental nor intentional, suggesting that for a substantial subset, the motivation or context for encountering content was ambiguous or difficult to categorize.

“Enactors” (287/567, 50.6%) in our sample were more likely to have deliberately sought out suicide-related content, whereas “ideators” (280/567, 49.4%) (ie, those who had experienced suicidal thoughts but never acted on them) were more likely to report encountering such content accidentally. Differences between these 2 groups in how they first accessed online material relating to suicide (ie, stumbling, seeking, or an unsure path) were statistically significant (*χ*²_2_=8.54; n=329; *P*=.01).

As the definition implies, stumblers were doing something else online before first encountering suicidal content. In follow-up questions, stumblers overwhelmingly reported either general browsing or looking at social media. The following 2 descriptions illustrate participants’ recollection of this first interaction:

*It was on a forum on Mumsnet, “top” stories are flagged- I was up in the early hours feeding my baby so I clicked without realizing what the post was about.*

[No gender given, >40 years]

*It was someone’s Facebook status.*

[Female, 25-34 years]

In contrast, follow-up questions for seeker*s* explored their reasons for initially seeking out suicide-related content. Participants often reported having multiple motivations. The most frequently reported reasons were a desire to hear about others’ experiences (47/76, 61.8%), not feeling understood by people offline (35/76, 46.1%), feeling able to talk more openly online (31/76, 40.8%), and not getting the support they needed offline (30/76, 39.5%). The following quotes highlight how many were looking for relatability, understanding, or shared narratives in moments of distress:

*I am still figuring out my feelings, and I don't know if I want to live or die and being able to navigate these thoughts with other people who know how I feel helps.*

[No gender given, 16-24 years].

*Reading threads by other people about things I can relate to makes me feel less alone. Talking about suicide and suicidal thoughts is taboo, often friends and family don’t want to hear it.*

[Female, 16-24 years]

Table 2 shows that the proportion of stumblers and seekers remains relatively stable over time, although intent does evolve for some. When asked about their current engagement with suicide-related content, as opposed to their first encounter, participants tended to report similar patterns of intent. For instance, of those who initially encountered content accidentally, the majority (95/121, 78.5%) continued to report accidental engagement. Likewise, most individuals who initially sought out content intentionally continued to do so (38/69, 55.1%). The large proportion who initially reported neither accidental nor intentional encounters continued to report similarly nondistinct engagement patterns over time (59/130, 45.4%).

**Table 2.** Comparing initial with current intent to view suicidal content (N=320)^a^.

Coming across suicide content for the first time was...	Coming across the content now is...		
	Accidental (n=156), n (%)	Intentional (n=77), n (%)	Neither (n=87), n (%)
Accidental (n=121)	95 (29.7)	12 (3.8)	14 (4.4)
Intentional (n=69)	17 (5.3)	38 (11.9)	14 (4.4)
Neither (n=130)	44 (13.8)	27 (8.4)	59 (18.4)

a Cell frequencies are shown with their percentages in brackets, reflecting the proportion of the total sample who answered both questions.

### Types of Content

The type of content that participants remembered seeing the first time was overwhelmingly descriptive user-generated content (155/351, 44.2%), with the second most common answer being visual user-generated content (43/351, 12.3%), in which users were sharing experiences of suicidal feelings or attempts. For example, 1 participant described the following sequence:

*I think I had just turned 13(ish) and was exploring YouTube/Tumblr and found some stuff that was a bit more intense than the self-harm content. I was curious to see if I would relate to it so carried on looking.*

[Female, 25-39 years]

*A mother shared her story about her son trying to commit suicide.*

[Male, >40 years]

*I feel like it was mentioned on the news in my last few years of primary school. So, I googled information because I was interested/ confused by the idea. Because of how google tracks your searches I feel like that’s why I saw posts about it on Instagram because of the algorithm.*

[No gender given, 16-24 years]

At a more granular level, the types of content that stumblers and seekers usually recall seeing online are notably different. Because these were optional, multiple response items, the number of responses varies by group and exceeds participant counts in some analyses. When comparing the 2 groups, stumblers were somewhat more likely to report seeing content on self-care, suicide prevention, seeing suicide stories in the news, or content from someone they know directly, as shown in Figure 1. By contrast, Mann-Whitney *U* testing indicated that seekers were more likely to report having seen information about methods (*U*=879.50; *z*=5.26; *P*<.001), academic articles (*U*=1302.50; *z*=5.05; *P*<.001), imagery or video depicting suicide (*U*=144.00; *z*=3.18; *P*=.001), and livestreaming of suicide attempts (*U*=16.00; *z*=2.57; *P*=.01), with all response rates illustrated in Figure 2.

*Any extreme violence in order to ground me about the realities of the fragility of a human being. I like to be reminded that it’s actually a lot easier to die than most people think.*

[Male, 25-39 years]

*In the last hour I just look at a few different websites talking about how to buy suicide kits, where suicide hotspots are near me, the most successful methods.*

[No gender given, 16-24 years]

**Figure 1.**
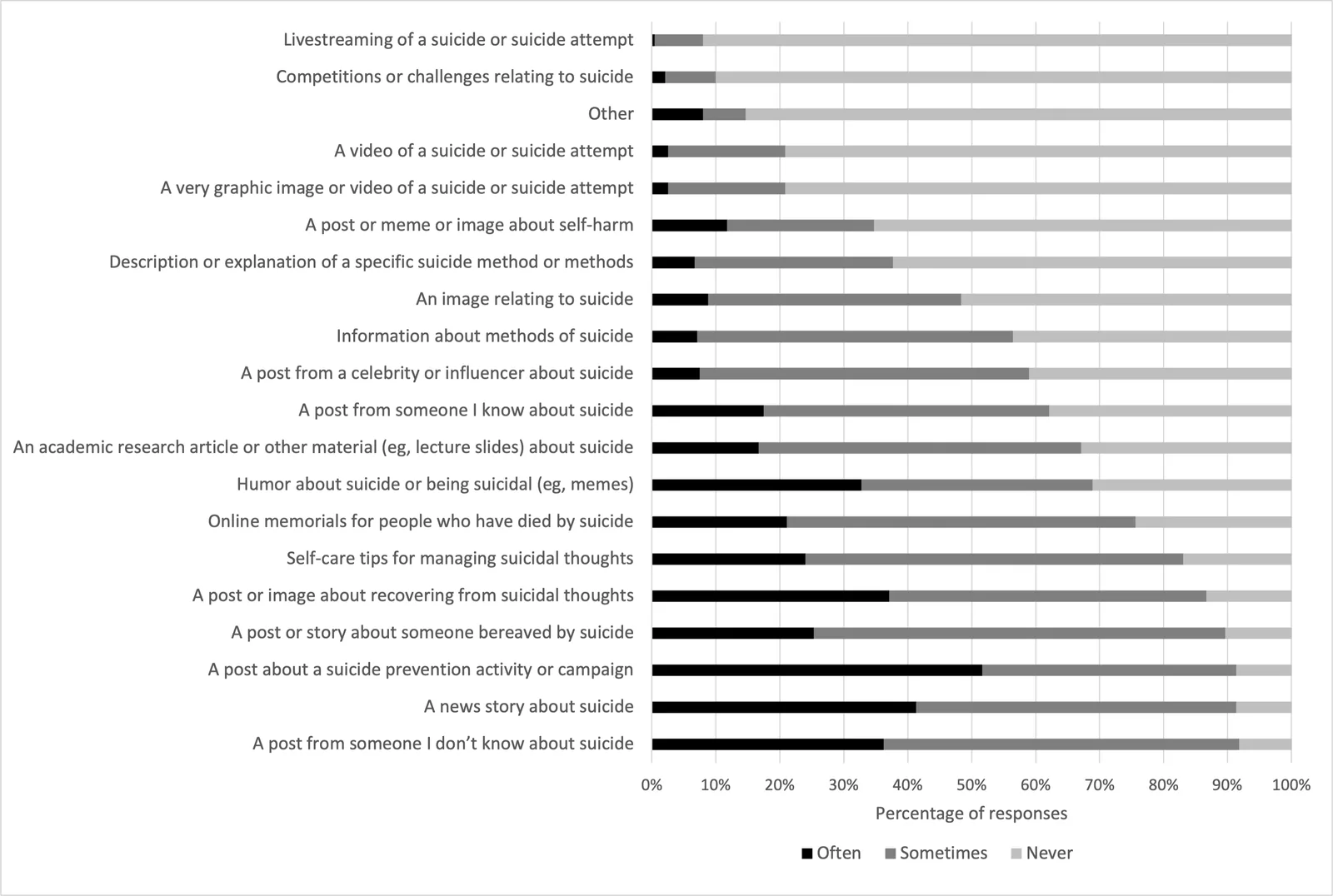
Frequency of encountering different types of suicide-related online content among stumblers (N=151). Percentages reflect the proportion of responses within each content category; participants could select multiple content types.

**Figure 2.**
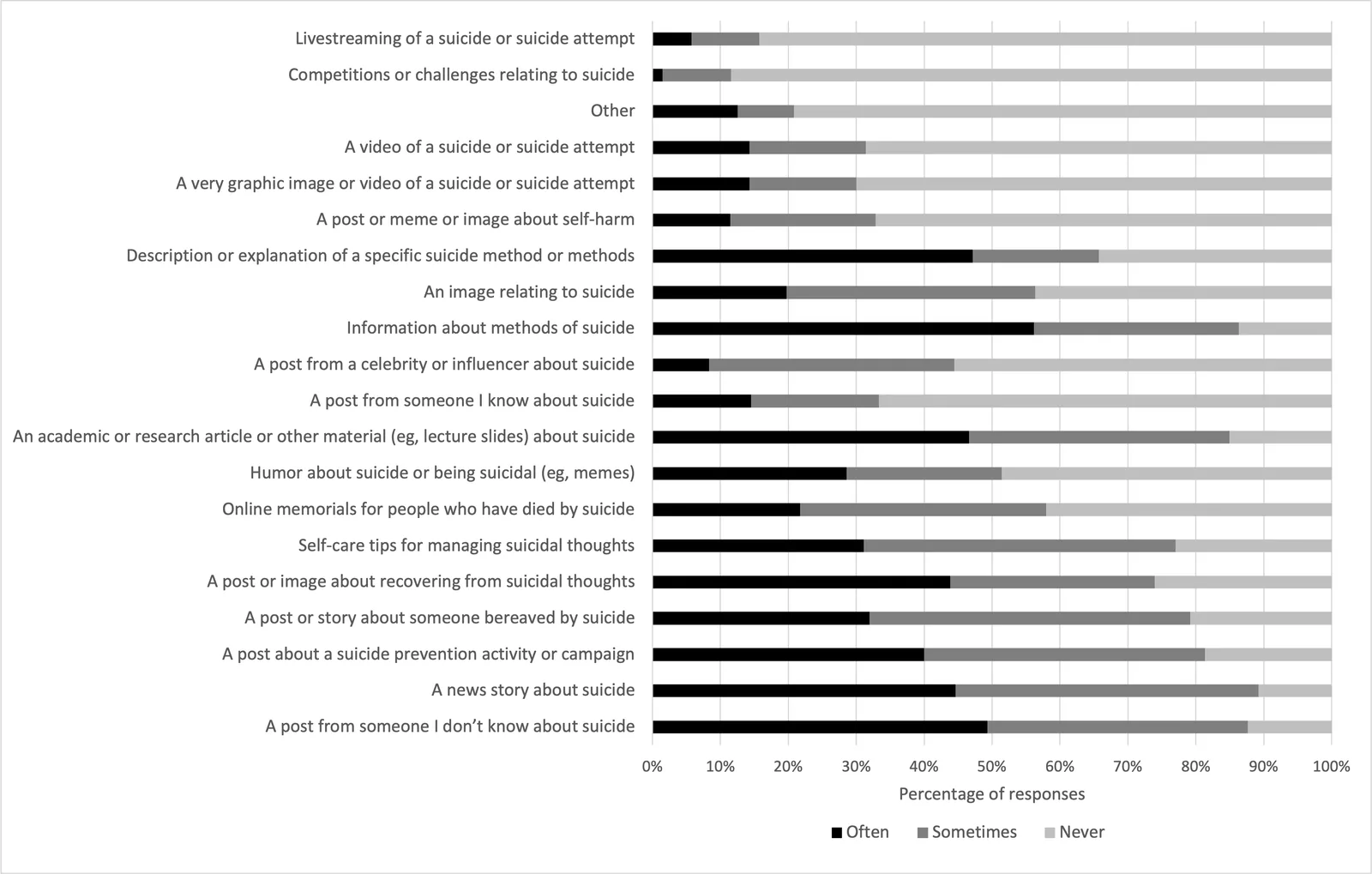
Frequency of encountering different types of suicide-related online content among seekers (N=76). Percentages reflect the proportion of responses within each content category; participants could select multiple content types.

### Response to Content Over Time

On first encountering suicide-related content, out of 276 participants, just over half (n=142, 51.4%) reported a negative emotional reaction, while only 1 in 10 (n=29, 10.5%) responded positively. Approximately 2 in 5 (n=105, 38.0%) described mixed reactions, such as a combination of curiosity and either positive or negative feelings. However, participants’ emotional responses appeared to shift over time. When asked about their most recent encounters with such content, participants reported reactions that differed significantly from those at first encounter (*χ*²_4_=65.67; n=276; *P*<.001). Responses to recent interactions were more likely to be positive or mixed, suggesting that perceptions of suicide-related content are not static, but likely evolve with context, experience, and emotional state. The quotes below illustrate the complex range of emotions communicated by participants when asked about seeing suicidal content:

*It doesn’t really make me feel any way. It makes me angry when people post about methods of suicide, especially online news stories and celebrities eg, on twitter you often see people talking about how celebrities killed themselves and I don't think this is appropriate.*

[Female, 16-24 years]

*Wanting to improve support for people.*

[Female, 16-24 years]

*It varies too much to specify depending on context.*

[Female, 25-39 years]

*Validated.*

[Female, 16-24 years]

Figure 3 shows the emergent hierarchy of emotional responses, ordered by frequency of reporting, with negative emotions dominating at the top. Notably, out of 352 participants, the most popular negative multicode responses were “upset” (n=141, 40.1%) and “unsettled” (n=168, 47.7%). The most popular positive responses were feeling “less alone” (n=74, 21.0%), “wanting more” (n=68, 19.3%), and a “sense of belonging” (n=47, 13.4%), suggesting a coherent pattern of responses oriented toward experiences of social connection, rather than discrete or unrelated positive effects.

When intent was used to analyze responses further, stumblers and seekers differed in their emotional responses to the suicide-related content they encountered (*χ*²_4_=44.71; n=352; *P*<.001). Those who stumbled upon the content were more likely to report negative emotional reactions, including feeling unsettled or shocked, whereas seekers were more likely to describe experiencing a sense of belonging, calm, or comforted:

*It depends what type of content. I generally find it ok as it is a person talking about their recovery. I don't ever see anything very graphic though*.

[No gender given, 16-24 years]

**Figure 3.**
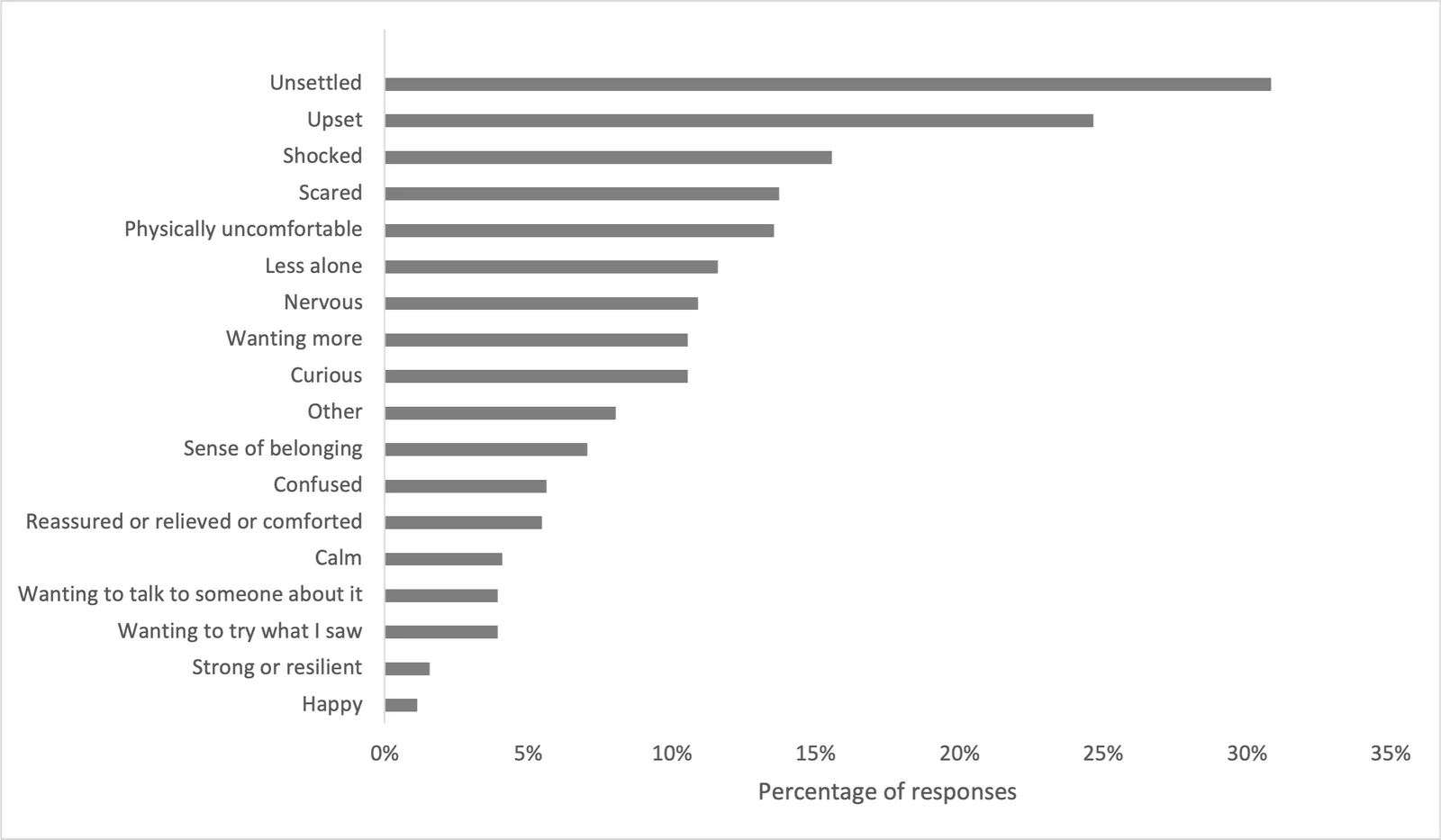
Emotional responses reported on first encounter with suicide-related online content (n=352; multiple responses permitted).

### Modes and Motivations of Engagement

For most participants, viewing suicide-related content represented only a small portion of their overall time online. However, for approximately 3% of participants, it made up most of their online activity.

Half of the participants reported actively liking or commenting on posts at least once (192/381, 50.4%), while the other half described themselves as passive consumers of content (189/381, 49.5%). Among the 176 participants, the reasons for active engagement included offering practical help and support (n=61, 34.7%), expressing solidarity (n=14, 7.9%), or for unclear reasons (n=38, 21.6%). Those who did not engage cited concerns about privacy, fear of making things worse, or the need to protect their own mental health.

Of the total sample, almost 1 in 4 (121/548, 22.1%) participants reported having posted suicide-related content themselves, whether about their own experiences or other material. The propensity to share such content about themselves online is not clearly correlated with seeker-stumbler categorization (*χ*²_6_=12.09; n=377; *P*=.06).

This is compared to around two-thirds (316/529, 59.7%) who discussed their own experiences relating to suicide offline, even if only rarely. There is a strong and statistically significant association between the frequency of discussing suicide offline and online (*χ*²_12_=103.84; n=528; *P*<.001). This result indicates that these 2 behaviors are not independent. Individuals who frequently talk about suicide offline are more likely to also talk about it online, and vice versa.

Nevertheless, some qualitative responses suggest that sharing online may be preferable for some. The most common reasons for sharing online were to vent personal feelings (32/106, 30.2%) or for unclear and uncertain reasons (19/106, 17.9%). One participant described the difficulties in sharing their feelings offline:

*I must be very careful when talking about suicide in person. I've made one serious attempt and been hospitalized many times. Now, if I bring up suicide everyone goes nuts.*

[Male, >40 years]

### Impact on Behavior

Out of 338 participants, almost one-third of participants (n=101, 29.9%) said that viewing suicide-related content had sometimes stopped them from attempting suicide, and a further 6.8% (n=23) of participants said it had often stopped them from making an attempt. Around a quarter (91/344, 26.5%) said that seeing or engaging with content exacerbated their suicidal feelings. Yet just over half (n=185, 53.8%) reported that seeing or engaging with content worsened their mood. Of these participants, some provided additional qualitative responses. They described being influenced by stories shared by those bereaved by suicide (13/75, 17.3%), accounts of failed attempts and associated risks (13/75, 17.3%), and content that linked to support services (9/75, 12%). One described how they were impacted by a story of a failed attempt:

*You see the danger/how difficult it may be and that it is terrifying and can cause permanent damage if you survive*.

[Female, 16-24 years]

Conversely, around one-fifth (68/338, 20.1%) said the content had contributed to a suicide attempt. At least for some, it seems that it might be less about feeling emotionally triggered as such, and more about “learning” and “storing” information about methods:

*It wasn’t that it triggered me, it’s just that I found out other people had ordered medication online for overdosing.*

[Female, 25-39 years]

For others, however, the trigger was more akin to a drive to replicate what they had found:

*If someone would share something that resonated with me (a quotation, song lyric), it usually reminded me how badly I felt. I remember also seeing how badly some people self-harmed/attempted suicide, and it made me feel like I wasn’t “doing it right.”*

[Female, 25-39 years]

## Discussion

### Principal Findings

This study contributes highly specific, first-hand insights into the complex role that suicide-related online content plays in the lives of individuals with lived experience of suicidality. Our findings add to a growing body of research challenging predominantly risk-focused accounts of suicide-related content. Rather, they suggest how an individual’s context, such as their intent and type of suicidality, can affect the impact of finding such content. In addition to potentially causing harm, such content can align closely with users’ preexisting emotional states and unmet needs, serving a reflective or validating role. The core finding is that content is perceived to reduce suicidality for some people, even preventing attempts at suicide, while simultaneously exacerbating suicidal feelings and facilitating attempts for others. This perspective raises urgent questions for traditional policy frameworks, including the UK’s OSA [14], which primarily emphasizes risk and harm through content removal strategies. Our findings suggest that policymakers need a nuanced understanding of online content’s role, particularly given the high prevalence of early and often accidental encounters among younger users.

Building on the work of Biddle et al [8], this study highlights the value of categorizing users as “stumblers” and “seekers” of suicide-related content. There were significant differences in emotional responses between these 2 groups, with “stumblers” frequently experiencing distress, and “seekers” more likely to find validation or comfort. Furthermore, a statistically significant link between suicidality type and engagement mode emerged; those who had attempted suicide (“enactors”) were more likely to seek content actively, while those with suicidal ideation but no suicidal actions typically encountered content unintentionally. Some “seekers” articulated their online activities as attempts to address offline deficiencies, such as inadequate social support or stigma surrounding open discussion. This suggests that seekers found online content during moments of crisis not because the content itself caused distress, but because it allowed them to navigate distress they were already feeling. This pattern reinforces longstanding evidence that online risks often reflect, rather than generate, preexisting vulnerabilities [15].

While this study supports a distinction between *stumblers* and *seekers*, our findings also point to the limitations of this binary. A substantial proportion of participants described their engagement as “neither accidental nor intentional.” Almost half of this group (59/130, 45.4%) maintained that ambiguity over time, suggesting that intent is not always easily articulated in this context. This may be due to the nature of algorithmic feeds, which blur the line between curation and agency [16,17], as well as the challenges individuals face in accurately identifying and articulating their own motivations and behaviors related to suicidality [18]. These findings underscore the need for more flexible conceptual models that recognize the fluid and often ambivalent ways people interact with suicide-related content.

Participants’ emotional reactions to suicide-related content were shown to develop significantly from initial encounters to more recent interactions. While initial reactions were predominantly negative, particularly for *stumblers*, later encounters tended to evoke more positive or mixed responses. This suggests an evolving relationship with online content over time, possibly influenced by factors such as familiarity with the content [19] and desensitization through repeated engagement, which may increase individuals’ psychological tolerance for distressing material and, in line with theoretical accounts of acquired capability [20,21], may increase psychological tolerance for distressing material through repeated exposure. Notably, while many users maintained consistent engagement patterns (stumblers remaining passive and seekers active), some transitioned from accidental to deliberate engagement. This shift points to a feedback loop where accidental encounters can evolve into intentional engagement (eg, [22]). It reflects the complex relationship between suicidality and online content use and highlights the need for longitudinal research to better understand how these patterns develop over time.

Our findings highlight the complex and ambivalent nature of suicide-related content. For some participants, it appeared to serve a protective function: a third reported that it had helped stop them from attempting suicide. For others, however, the content was linked to increased risk, with 1 in 5 perceiving the content as having contributed to a suicide attempt. Rather than reflecting a contradiction, these outcomes illustrate the diverse ways individuals engage with such material, shaped by their intent, emotional state, and the context in which they encounter it. If users are searching for suicide-related content because they already feel suicidal, then the content is arguably not the origin—it is a mirror. As a result, removing this content could have unintended consequences for those who reported relying on it as a source of comfort or validation. Treating all suicide-related content as inherently harmful may overlook the critical role it plays in meeting the emotional needs or offering support to certain vulnerable users.

### Implications for Policy and Future Research

For the vast majority, viewing this content makes up a small percentage of their time spent online. These findings resist the idea of content that pulls users into harmful spirals through compulsive, addictive, or manipulative properties (eg, [23]). Instead, despite being part of more generalized browsing, they point to selective, casual engagement, especially among those already in crisis or trying to understand themselves.

The findings call for more nuanced regulatory frameworks that recognize the complex roles suicide-related content can play, particularly for those in moments of acute crisis. Policies should seek to understand and prioritize user intent and context over blanket content removal strategies, which may act to “re-silence” those who lack support [23]. Future research should further investigate longitudinal patterns of engagement, the role of content delivery, and how users’ states, for example, type of suicidality, interact with online content. Additionally, exploring diverse populations and platform-specific dynamics can further enhance our understanding and guide effective policy and intervention strategies. For example, aligning support interventions with users’ likely emotional states and motivations may offer effective routes for users to seek help. These approaches could help tailor support without relying on overt disclosures, which many individuals in crisis may be unable or unwilling to make [24]. From a clinical perspective, exploring young people’s online and social media use as part of risk and psychosocial assessment may provide important insight into their emotional state and available support systems. Monitoring changes in online engagement over time could also inform formulation and help clinicians identify shifts in risk or opportunities to intervene.

### Limitations

The study has several limitations. The sample consisted of self-selected individuals with significant lived experience of suicidality and an unrepresentatively high proportion of transgender participants, limiting the study’s representativeness of the broader population. Additionally, those currently experiencing acute suicidal crises were discouraged from taking part, thereby potentially underrepresenting perspectives on immediate harms and benefits of content engagement. The retrospective design of the survey also likely introduces recall biases, which may be significant given the topic and sample. Some survey questions had a relatively low number of responses, which may limit the reliability of subgroup comparisons. Additionally, while content category coding was carefully conducted with steps taken to minimize subjectivity, variation in interpretation remains a possible source of bias. Despite these limitations, the depth of qualitative insights gained provides valuable direction for future research and policy.

### Conclusions

This research highlights the complex and often ambivalent role of suicide-related online content. It emphasizes the importance of user intent, emotional context, and evolving relationships with online content. To effectively address suicide-related harms and harness potential benefits, policy and clinical responses must acknowledge the reflective and contextual nature of online interactions, ensuring supportive environments that validate rather than silence users’ experiences. Therefore, the findings caution against policies that rely on content removal as a protective strategy, without recognizing the potential value such content holds for some individuals.

## Supplementary material

10.2196/92693 Multimedia Appendix 1 Full study survey questionnaire.

10.2196/92693 Multimedia Appendix 2 Content analysis codebook.

## Abbreviations

DSIT: Department for Science, Innovation and Technology (UK)
NHS: National Health Service
OSA: Online Safety Act
