# Supplementary material for: Navigating Suicide-Related Online Content in Individuals With Lived Experience of Suicidality: Cross-Sectional Survey
Source: J Med Internet Res. 2026 Oct 20;28:e92693. doi: 10.2196/92693 (PMC13685424; doi:10.2196/92693)
Supplement: Supplementary file 1 [file jmir-v28-e92693-s001.docx]

**Appendix 1**

Full Survey

*Title: Help us learn from your experiences of seeing or posting online content about suicide and self-harm*

[SECTION A. SOCIO-DEMOGRAPHIC QUESTIONS]

*To help us understand the context of your experiences, please tell us a bit about yourself. This also helps us understand how people’s online and offline experiences can vary across age, gender and other characteristics (for example where you live or whether you are working or studying), and therefore what forms of support are best suited to different groups.*

*All of these questions are optional – you may skip any that you would prefer not to answer.*

1. How old are you?

- Under 16 [screen out]
- 16-19
- 20-24
- 25-29
- 30-39
- 40-49
- 50-59
- 60+
- Prefer not to say

2. This question is about your gender identity. Do you identify as:

- woman/girl
- man/boy
- transwoman/transgirl
- transman/transboy
- non-binary/genderqueer/agender/gender fluid
- don’t know
- prefer not to say
- other [please specify: ___ ]

3. What was your sex assigned at birth?

- female
- male
- prefer not to say

4. Which of the following best describes your sexual orientation?

- Asexual
- Bisexual
- Gay or lesbian
- Heterosexual/straight
- Queer
- Questioning/unidentified
- Prefer not to say
- Other [please specify: ___ ]

5. How do you describe your ethnicity? [Please tick against one of the following]:

| Asian or Asian British  Bangladeshi  Chinese  Indian  Pakistani  Other: ______  Black or Black British  African  Caribbean  Other: _______ | Mixed/ Multiple Ethnic Groups  White/Asian  White/Black African  White/Black Caribbean  White/Arab  Other: ______  White  Welsh/ English/ Scottish/ Northern Irish/  British  Irish  Gypsy or Irish Traveller  Other: _______ |
| --- | --- |

If you prefer to describe your ethnicity in any other way, please specify: _________

6. Do you have a religion, faith or belief?

Yes/No (if yes ->)

- Buddhist
- Christian
- Hindu
- Jewish
- Muslim
- Sikh
- Pagan
- Other religious or spiritual belief (Please specify if you wish): _______________________
- Prefer not to say

7. Do you live in the UK?

- No (Please specify if you wish.......) -> screen out
- Yes ->
- East of England/East Anglia
- East Midlands
- London
- North East
- North West
- Scotland
- South East (excluding London)
- South West
- West Midlands
- Yorkshire and the Humber
- Wales
- Scotland
- Northern Ireland

8. Do you live in an urban or rural area?

- Urban/city/town
- Rural/countryside/village
- Other: ______

9. Which of the following best describes your current status?

- Working full-time
- Working part-time
- Studying
- Looking after my home or family
- Looking for a job
- Unable to work because of illness or disability
- Full-time carer
- Retired
- Other: _______________

10. Does your current or previous occupation involve working with people in distress or experiencing mental health difficulties? (E.g. you may have trained or worked as a mental health professional, volunteered for a mental health charity or worked with young people experiencing emotional difficulties)

No/Yes (-> Please specify if you wish: _________)

11. What is the highest level of education you have achieved?

No formal qualifications

GCSEs/ O Levels or international equivalent

A Levels or international equivalent

Bachelor's Degree

Master's Degree/ Professional qualification

Doctoral degree

Other:

12. Which statement best describes your current living arrangements? [please tick as many as apply]

- Living alone
- Living with parent/s
- Living with sibling/s
- Living with partner
- Living with children
- Living with other family members
- Living with friends
- Living in shared accommodation
- Other (please specify)

[SECTION B. EXPERIENCES OF SELF-HARM AND MENTAL HEALTH DIFFICULTIES/SERVICES]

*This next section includes some questions about your experiences (if any) of self-harm and suicide attempts, and about your physical and mental health more generally. Once again, please skip any questions you would prefer not to answer.*

In this survey, we define ‘self-harm’ as: “any deliberate act of self-poisoning or self-injury *without* suicidal intent. This excludes accidents, substance misuse and eating disorders.”

1. Have you ever thought about harming yourself without wanting to take your own life (i.e. self-harm but not as a suicide attempt)?

| Never  [go to Q.3] | Once | Between 2 and 4 times | Five times or more |
| --- | --- | --- | --- |

2. Have you ever self-harmed without wanting to take your own life?

| Never  [go to Q.3] | Once | Between 2 and 4 times | Five times or more |
| --- | --- | --- | --- |

2.1 Do you have a main form or method of self-harm? __________

2.2 How old were you when you first self-harmed? (Please give approximate age) _______

2.3 When was the last time you self-harmed?

- Within the last week
- Within the last month
- Within the last year
- Over a year ago

2.4 Do you feel that you have stopped self-harming?

- Yes
- No
- I don’t know

3. Have you ever had thoughts of ending your life?

- No [Skip to Q5]
- Yes - In the last six months
- Yes - Between 6 and 12 months ago
- Yes - 1-2 years ago
- Yes- 2-5 years ago
- Yes - Over 5 years ago
- Prefer not to say

4. Have you ever attempted suicide?

| Never  [Skip to Q5] | Once | Between 2 and 4 times | Five times or more |
| --- | --- | --- | --- |

4.1 How old were you when you first attempted suicide? (Please give approximate age)

4.2 When did you last attempt suicide?

- In the last six months
- Between 6 and 12 months ago
- 1-2 years ago
- 2-5 years ago
- Over 5 years ago
- Prefer not to say

5. Have any of your family or close friends ever self-harmed, without wanting to die? Yes/No

6. Have any of your family or close friends ever attempted or died by suicide? Yes/No

7. Do you know anyone else who has self-harmed, attempted or died by suicide? Yes/No Please tell us more if you wish

8. Have you ever received any support or treatment for your mental health from the following? [please tick as many as apply]

- GP or family doctor
- NHS mental health services – as an inpatient
- NHS mental health services – as an outpatient
- School/University/work therapist or counsellor
- Private therapist/counsellor
- Charity (e.g. Samaritans, Mind)
- Other voluntary or community support
- Online therapist/mental health app
- Other (please specify)

9. Do you have any serious physical health conditions? Yes/No

Please tell us more if you wish

10. Do you have any neurodevelopmental difficulties or conditions (e.g. autism, ADHD)? Yes/No

Please specify:

[SECTION C. ONLINE BEHAVIOUR AND EXPOSURE TO SELF-HARM CONTENT]

*Please tell us a bit about your online activities and your experiences (if any) of seeing, sharing or posting online content about (non-suicidal) self-harm.*

1. How many hours per day, on an average day, do you spend online (e.g. browsing the Internet, on social media, gaming, reading online news - but excluding any online learning or work-related activities such as email or Zoom meetings)?

- Less than one hour
- Between 1 and 3 hours
- 4-6 hours
- 7 hours or more

2. Which site or sites do you access on a regular (at least weekly) basis? [please tick as many as apply]

- Tiktok
- Twitter
- Facebook
- Instagram
- Snapchat
- Youtube
- Pinterest
- Linkedin
- Reddit
- Tumblr
- Tellonym
- Gaming sites
- Gambling sites
- Support websites (e.g. Samaritans, Mind)
- Other: _______

3. Do you have more than one user account for any of these sites? Yes/No

Please tell us more about this if you wish (e.g. which sites do you have multiple user accounts for? Why?)

4. Are there specific online forums or closed online groups you access on a regular basis? Yes/No

Please specify:

5. How often, if ever, have you seen a post or other online content about someone who intentionally harms themselves (without wanting to die) (self-harm content)?

| Never  [Skip to Q11] | Once  [Go to Q6 then skip to Q11] | Between 2 and 4 times | Five times or more |
| --- | --- | --- | --- |

6. Thinking about the FIRST time you came across any self-harm content online:

Please tell us in your own words what you saw and where (e.g. you may have seen a video about self-harm on YouTube or a post on Facebook about someone talking about wanting to self-harm or showing any self-inflicted scars or injuries)

- How old were you at the time? (please give approximate age)
- Coming across this content was
- Accidental
- Intentional
- I’m not sure
- I don’t know/can’t remember
- What were you doing at the time:
- General browsing
- Gaming
- Looking at my social media feed/timeline
- Reading the news
- Searching information or help for myself
- Searching information or help for somebody else
- I was on a site or forum about eating disorders
- I was on a site or forum about suicide
- I was on a site or forum about self-harm
- I was on a site or forum about mental health problems (e.g. depression, anxiety)
- Other:
- Can you recall what search/searches or hashtags took you to this content?
- Did somebody else show you or send you this content (including by tagging you in or sharing it with you in a private message)? Yes/No

If yes - Please tell us more about this if you wish.

- When you FIRST saw self-harm content online, how did it make you feel? [tick as many as apply]
- Confused
- Curious
- Unsettled
- Happy
- Stronger/more resilient
- Nervous
- I felt a sense of belonging
- Scared
- Shocked
- Calm
- Physically uncomfortable
- Less alone
- Upset
- Reassured/relieved/comforted
- Wanting to know or see more
- Wanting to try out what I saw
- Wanting to talk to someone about what I saw
- Other. Please specify:

At the time:

- Had you ever self-harmed? Yes [Skip Q]/No
- Had you ever had thoughts of self-harm? Yes [Skip Q]/No
- Were you aware of self-harm? Yes/No

7. Thinking about the LAST time you came across any self-harm content online: [Skip question if ‘once’ to Q5]

How long ago was this?

- In the last week
- In the last month
- Over a month ago
- Over a year ago

Please tell us in your own words what you saw and where

8. When I see online content about self-harm, this is:

1. Always/mostly accidental (e.g. shared with me by someone else)

2. Often accidental

3. I’m not sure/neither accidental nor intentional

4. Often intentional

5. Always/ mostly intentional

8.1 [if answered 1-3 above; then skip to 8.3]: What sort of self-harm content do you usually come across online?

| A post from someone I know about their self-harm | Often/Sometimes/Never |
| --- | --- |
| A post from someone I don’t know about their self-harm | Often/Sometimes/Never |
| A post from a celebrity or influencer about self-harm | Often/Sometimes/Never |
| An image of self-harm | Often/Sometimes/Never |
| A video of self-harm | Often/Sometimes/Never |
| Livestreaming of self-harm | Often/Sometimes/Never |
| A very graphic description or image/video of self-harm | Often/Sometimes/Never |
| Competitions or challenges relating to self-harm | Often/Sometimes/Never |
| Information about methods of self-harm  A very detailed description/explanation of a specific method of self-harm | Often/Sometimes/Never  Often/Sometimes/Never |
| A news story about self-harm | Often/Sometimes/Never |
| An academic/research article or other material (e.g. lecture slides) about self-harm | Often/Sometimes/Never |
| A post to raise awareness of self-harm or encouraging people to seek help (e.g. from a charity) | Often/Sometimes/Never |
| A post or image about self-harm recovery | Often/Sometimes/Never |
| Humour about self-harm (e.g. memes) | Often/Sometimes/Never |
| Self-care tips for self-harm | Often/Sometimes/Never |
| A post/meme/image about self-harm that somebody else sent/shared/tagged me in | Often/Sometimes/Never |
| Other. Please specify: | Often/Sometimes/Never |

OR 8.2 [if answered 4-5 to Q8]: What sort of self-harm content do you usually seek or look out for online?

| A post from someone I know about their self-harm | Often/Sometimes/Never |
| --- | --- |
| A post from someone I don’t know about their self-harm | Often/Sometimes/Never |
| A space to discuss my own self-harm | Often/Sometimes/Never |
| A space to post images of my own self-harm  Something to distract me from my own self-harm thoughts/urges | Often/Sometimes/Never  Often/Sometimes/Never |
| A post from a celebrity or influencer about self-harm | Often/Sometimes/Never |
| An image of self-harm | Often/Sometimes/Never |
| A video of self-harm | Often/Sometimes/Never |
| Livestreaming of self-harm | Often/Sometimes/Never |
| A very graphic description or image/video of self-harm | Often/Sometimes/Never |
| Competitions or challenges relating to self-harm | Often/Sometimes/Never |
| Information about methods of self-harm  A very detailed description/explanation of a specific method of self-harm | Often/Sometimes/Never  Often/Sometimes/Never |
| An academic/research article or other material (e.g. lecture slides) about self-harm | Often/Sometimes/Never |
| Information or links to support for self-harm | Often/Sometimes/Never |
| A post or image about self-harm recovery | Often/Sometimes/Never |
| Humour about self-harm (e.g. memes) | Often/Sometimes/Never |
| Self-care tips for self-harm | Often/Sometimes/Never |
| Other. Please specify: | Often/Sometimes/Never |

What motivates you to seek online content about self-harm? Please tick all that apply

- I don’t get the support I need offline
- I feel I can talk more openly about my experiences online
- I feel like people offline don’t understand what I’m experiencing
- I can get immediate support from other users when I am having thoughts about hurting myself
- I want to hear about other people’s experience of self-harm or suicide
- I find it complements or adds to the support I get offline
- Curiosity
- I like looking at images of self-harm
- I want to support others with their self-harm
- Other: ________________________

8.3 Usually, how does seeing self-harm content online make you feel now? [tick as many as apply]

- Confused
- Curious
- Unsettled
- Happy
- Stronger/more resilient
- Nervous
- I feel a sense of belonging
- Scared
- Shocked
- Calm
- Physically uncomfortable
- Less alone
- Upset
- Reassured/relieved/comforted
- Wanting to know or see more
- Wanting to try out what I see
- Wanting to talk to someone about what I see
- Other. Please specify:

9. Do you follow particular groups or hashtags relating to self-harm?

Please tell us more if you wish________

10. Have you ever responded to other people’s posts (including images and videos) about self-harm (e.g. liked or commented)?

| Never | Once | Between 2 and 4 times | Five times or more |
| --- | --- | --- | --- |

Please tell us more about this (E.g. when or why are you more likely to respond to online content about self-harm? Why/when do you prefer not to?)

11. Have you ever posted your own experiences or images about self-harm, or any other online content about self-harm?

| Never  [Skip to Q12, or section D if also never at Q5] | Once | Between 2 and 4 times | Five times or more |
| --- | --- | --- | --- |

Please tell us more about this if you wish (e.g. what sort of content have you posted? Why?):

- Do you also discuss these things offline? always->never

Please tell us more about this if you wish (e.g. who do you tend to discuss these things with? When?): ______________

12. Have you ever shared or re-posted someone else’s online content about self-harm?

| Never  [Skip to Q13] | Once | Between 2 and 4 times | Five times or more |
| --- | --- | --- | --- |

Please tell us more about this if you wish (e.g. what sort of content have you shared/re-posted? Why? Did you share this with a particular person or group, or more widely/publicly?):

13. How much of your time online (excluding online learning or work) do you normally spend intentionally viewing/posting/responding to online content about self-harm?

- None
- Very little/Less than 10%
- A little/between 10 and 30%
- Less than half
- More than half
- All or almost all my time online

14. What time or times of day do you normally post or intentionally view online content about self-harm? [please tick all that apply]

- Morning
- Afternoon
- Evening
- Night

15. Overall, seeing, sharing or posting online content about self-harm [5-point sliders]

a. Improves my mood -> worsens my mood

b. Stops me self-harming -> makes me want to self-harm

[if 1-2 to 15.b, then skip to 15.e]

Please tell us a bit more about this. What sort of online content or interactions have stopped you from self-harming?

[if 3 to 15.b, skip to 15.e]

[If 4-5 to 15.b] 13.c: Do you usually then act of these thoughts? Yes/no

If no: 15.d Please tell us a bit more about this. E.g., what content do you find triggers your self-harm thoughts or urges? When has seeing, sharing or posting something online triggered you in this way? What helps you not to act on these urges?

If yes [or responded 1-3 to 15.b]:

15.e As a consequence of seeing self-harm content online, have you ever performed the same (or very similar) self-harm? [never – sometimes – often]

15.f As a consequence of seeing self-harm content online, have you ever self-harmed more or more severely? [never – sometimes – often]

If sometimes or often to either 15.e or f: Please tell us a bit more about this. What content do you find triggers your self-harm? When has seeing, sharing or posting something online triggered your own self-harm?

[SECTION D. EXPOSURE TO SUICIDE CONTENT]

*In the next section we’d like to ask you some questions about your experiences (if any) or seeing, sharing or posting online content about suicide.*

1. How often, if ever, have you seen a post or other online content about suicide, including of someone contemplating, planning or attempting to take their life (suicide content)?

| Never  [Skip to Q7] | Once | Between 2 and 4 times | Five times or more |
| --- | --- | --- | --- |

2. Thinking about the FIRST time you came across any suicide content online:

Please tell us in your own words what you saw and where:

- How old were you at the time? (please give approximate age)
- Coming across this content was
- Accidental
- Intentional
- I’m not sure
- I don’t know/can’t remember
- What were you doing at the time:
- General browsing
- Gaming
- Looking at my social media feed/timeline
- Reading the news
- Searching information or help for myself
- Searching information or help for somebody else
- Researching a specific suicide method or location
- I was on a site or forum about eating disorders
- I was on a site or forum about (non-suicidal) self-harm
- I was on a site or forum about suicide
- I was on a site or forum about mental health problems (e.g. depression, anxiety)
- Other:
- Can you recall what search/searches or hashtags took you to this content?
- Did somebody else show you or send you this content (including by tagging you in or sharing it with you in a private message)? Yes/No

Please tell us more about this if you wish.

- When you FIRST saw suicide content online, how did it make you feel? [tick as many as apply]
- Confused
- Curious
- Unsettled
- Happy
- Stronger/more resilient
- Nervous
- I felt a sense of belonging
- Scared
- Shocked
- Calm
- Physically uncomfortable
- Less alone
- Upset
- Reassured/relieved/comforted
- Wanting to know or see more
- Wanting to try out what I saw
- Wanting to talk to someone about what I saw
- Other. Please specify:

3. Thinking about the LAST time you came across any suicide content online: [Skip if ‘once’ at Q1 and go to Q5]

How long ago was this?

- In the last week
- In the last month
- Over a month ago
- Over a year ago

Please tell us in your own words what you saw and where

4. When I see online content about suicide, this is:

1. Always/mostly accidental (e.g. shared with me by someone else)

2. Often accidental

3. I’m not sure/neither accidental nor intentional

4. Often intentional

5. Always/ mostly intentional

4.1 [if answered 1-3 above; then skip to 4.3]: What sort of suicide content do you usually come across online?

| A post from someone I know about suicide | Often/Sometimes/Never |
| --- | --- |
| A post from someone I don’t know about suicide | Often/Sometimes/Never |
| A post from a celebrity or influencer about suicide | Often/Sometimes/Never |
| An image relating to suicide | Often/Sometimes/Never |
| A video of a suicide or suicide attempt | Often/Sometimes/Never |
| Livestreaming of a suicide or suicide attempt | Often/Sometimes/Never |
| A very graphic image/video of a suicide or suicide attempt | Often/Sometimes/Never |
| Information about methods of suicide  A very detailed description/explanation of a specific suicide method or methods | Often/Sometimes/Never  Often/Sometimes/Never |
| A news story about suicide | Often/Sometimes/Never |
| An academic/research article or other material (e.g. lecture slides) about suicide | Often/Sometimes/Never |
| Competitions or challenges relating to suicide | Often/Sometimes/Never |
| A post about a suicide prevention activity or campaign (e.g. from a charity encouraging people to seek help) | Often/Sometimes/Never |
| A post or image about recovering from suicidal thoughts | Often/Sometimes/Never |
| Humour about suicide/being suicidal (e.g. memes) | Often/Sometimes/Never |
| A post or story about someone bereaved by suicide | Often/Sometimes/Never |
| Online memorials for people who have died by suicide | Often/Sometimes/Never |
| Self-care tips for managing suicidal thoughts | Often/Sometimes/Never |
| A post/meme/image about self-harm that somebody else sent/shared/tagged me in | Often/Sometimes/Never |
| Other. Please specify: | Often/Sometimes/Never |

OR 4.2 [if answered 4-5 to Q4]: What sort of suicide content do you usually seek or look out for online?

| A post from someone I know about suicide | Often/Sometimes/Never |
| --- | --- |
| A post from someone I don’t know about suicide | Often/Sometimes/Never |
| A post from a celebrity or influencer about suicide | Often/Sometimes/Never |
| A space to discuss my own suicidal thoughts/plans/attempts | Often/Sometimes/Never |
| Something to distract me from my own suicidal thoughts/plans/attempts | Often/Sometimes/Never |
| An image relating to suicide | Often/Sometimes/Never |
| A video relating to suicide | Often/Sometimes/Never |
| Livestreaming of a suicide or suicide attempt | Often/Sometimes/Never |
| A very graphic image/video of a suicide or suicide attempt  Information about methods of suicide  A very detailed description/explanation of a specific suicide method or methods | Often/Sometimes/Never  Often/Sometimes/Never  Often/Sometimes/Never |
| A news story about suicide | Often/Sometimes/Never |
| An academic/research article or other material (e.g. lecture slides) about suicide | Often/Sometimes/Never |
| Information or links to support for suicidal thoughts | Often/Sometimes/Never |
| Competitions or challenges relating to suicide | Often/Sometimes/Never |
| A post or image about recovering from suicidal thoughts | Often/Sometimes/Never |
| Humour about suicide/being suicidal (e.g. memes) | Often/Sometimes/Never |
| A post or story about someone bereaved by suicide | Often/Sometimes/Never |
| Online memorials for people who have died by suicide | Often/Sometimes/Never |
| Self-care tips for managing suicidal thoughts | Often/Sometimes/Never |
| Other. Please specify: | Often/Sometimes/Never |

What motivates you to seek online content about suicide? Please tick all that apply

- I don’t get the support I need offline
- I feel I can talk more openly about my experiences online
- I feel like people offline don’t understand what I’m experiencing
- I can get immediate support from other users when I am having thoughts about taking my own life
- I want to hear about other people’s experience of self-harm or suicide
- I find it complements or adds to the support I get offline
- Curiosity
- Other: ________________________

4.3 Usually, how does seeing online content about suicide make you feel now? [tick as many as apply]

- Confused
- Curious
- Unsettled
- Happy
- Stronger/more resilient
- Nervous
- I feel a sense of belonging
- Scared
- Shocked
- Calm
- Physically uncomfortable
- Less alone
- Upset
- Reassured/relieved/comforted
- Wanting to know or see more
- Wanting to try out what I see
- Wanting to talk to someone about what I see
- Other. Please specify:

5. Do you follow particular groups or hashtags relating to suicide?

Please tell us more if you wish________

6. Have you ever responded to other people’s posts (including images and videos) about suicide (e.g. liked, commented)?

| Never | Once | Between 2 and 4 times | Five times or more |
| --- | --- | --- | --- |

Please tell us more about this. E.g. when or why are you more likely to respond to online content about suicide? Why/when do you prefer not to?

7. Have you ever posted your own experiences or images relating to suicide, or any other online content about suicide?

| Never  [Skip to Q8 or section E if also never at Q1] | Once | Between 2 and 4 times | Five times or more |
| --- | --- | --- | --- |

Please tell us more about this if you wish (e.g. what sort of content have you posted? Why?):

- Do you also discuss these things offline? always->never

Please tell us more about this if you wish (e.g. who do you tend to discuss these things with? When?): ______________

8. Have you ever shared or re-posted someone else’s online content about suicide?

| Never  [Skip to Q9] | Once | Between 2 and 4 times | Five times or more |
| --- | --- | --- | --- |

Please tell us more about this if you wish (e.g. what sort of content have you shared/re-posted? Why? Did you share this with a particular person or group, or more widely/publicly?):

9. Have you ever researched a specific suicide method or location online?

| Never [go to Q10] | Once | Between 2 and 4 times | Five times or more |
| --- | --- | --- | --- |

Please tell us more about this if you wish. (e.g. What sort of site or sites did you access? Did you find the information you were looking for? Did you interact with other online users as part of this process? What was the impact of this? Did you use the information you obtained in any way?)

10. How much of your time online (excluding online learning or work) do you normally spend intentionally viewing/posting/responding to online content about suicide?

- None
- Very little/Less than 10%
- A little/between 10 and 30%
- Less than half
- More than half
- All or almost all my time online

11. What time or times of day do you normally post or intentionally view online content about suicide? [please tick all that apply]

- Morning
- Afternoon
- Evening
- Night

12. Overall, seeing, sharing or posting online content about suicide [5 point sliders]

a. Improves my mood -> worsens my mood

b. Makes me want to live -> makes me want to die

c. Has viewing online content about suicide ever stopped you from attempting suicide? [never – sometimes – often]

[if sometimes or often: Please tell us a bit more about this. What sort of online content or interactions have helped you in this way?]

d. As a consequence of seeing suicide content online, have you ever attempted suicide in the same or a very similar way (e.g. using the same method and/or location)? [never – sometimes – often]

[If sometimes or often: Please tell us a bit more about this. What kind of content has triggered you in this way? When has seeing, sharing or posting something online triggered or influenced your own suicide attempt?]

[SECTION E. SELF-HARM AND SUICIDE CONTENT]

*Finally, thinking about BOTH self-harm and suicide online content…*

1. What, if anything, do you do to avoid online content about suicide and self-harm that you might find disturbing or triggering: _________

2. How helpful do you find the following things online: (very unhelpful – very helpful)

- Self-care and wellbeing tips
- Stories of hope and recovery
- Campaigns or messages to raise awareness of self-harm or suicide
- People sharing their own experiences of self-harm or suicide
- Being able to share my own experiences of self-harm or suicide [add n/a option]
- People sharing graphic descriptions, images or videos of self-harm or suicide
- Humour about suicide or self-harm
- Trigger warnings
- Being able to report, block or unfollow posts or hashtags that I find upsetting or triggering
- Being able to make anonymous forum posts
- People sharing information or images about self-harm or suicide methods
- Sites or groups that are moderated (e.g. by a therapist or counsellor)
- Sites or groups that are not moderated
- Online spaces where suicide and self-harm content is banned
- Signposting to support options
- Being able to access immediate online support (e.g. online chats, therapy or counselling)
- Other:

3. Have you ever tried to access specific suicide and self-harm content but found this to be blocked/unavailable? Yes/No

Please tells us more about your experience of this if you wish (e.g. what did you do instead? How did it make you feel?)

4. Have you ever been reported, blocked or had content deleted for something you posted or shared online about suicide or self-harm? Yes/No [build conditionality so only asked if applicable]

Please tell us more about this. (e.g. How did it make you feel? Did you post the information elsewhere?)

5. Thinking about online content about suicide and self-harm:

- What would you like to see more of?
- What would you like to see less of?

6. Please use this space to record any additional comments about this (e.g. are there any important questions/areas we didn’t ask you about? Any other thoughts or experiences you’d like to share?)
