# Supplementary material for: Navigating Suicide-Related Online Content in Individuals With Lived Experience of Suicidality: Cross-Sectional Survey
Source: J Med Internet Res. 2026 Oct 20;28:e92693. doi: 10.2196/92693 (PMC13685424; doi:10.2196/92693)
Supplement: Supplementary file 2 [file jmir-v28-e92693-s002.docx]

**Appendix 2.**

Content Analysis Codebook

Q89 Has viewing online content about suicide ever stopped you from attempting suicide? Q 89a Please tell us a bit more about this. What sort of online content or interactions have helped you in this way?

Types of content/interactions:

1. Links to support and helplines

2. Recovery stories or lived experience accounts

3. Methods information

4. Peer support

5. Stories from bereaved family and friends

6. Motivational quotes

7. Online memorials

8. Website with reasons to live/information on why you shouldn’t attempt suicide

9. All types of suicide content

10. Tips and distraction techniques

11. Other

12. Not specified/unclear

13. Not relevant

14. Content or interactions that create a sense of belonging/community/not being alone

15. Awareness posts

Q90 As a consequence of seeing suicide content online, have you ever attempted suicide in the same or a very similar way (e.g. using the same method and/or location)? Q 90a Please tell us a bit more about this (e.g. what kind of content has triggered you in this way? When has seeing, sharing or posting something online triggered or influenced your own suicide attempt?)

Type of content/interactions:

1. Information about suicide methods

2. General posts or articles about suicide

3. ‘Recovery’ posts with methods information

4. Stories of others’ suicide

5. Other

6. Not specified/unclear

7. Not relevant (as did not attempt suicide)

8. Fictional media representations of suicide

9. Information about suicide hotspots

Q66a Thinking about the FIRST time you came across any suicide content online, please tell us in your own words what you saw and where?

Where:

1. YouTube

2. Twitter

3. Tumblr

4. TikTok

5. Facebook

6. Instagram

7. Reddit

8. Pinterest

9. Google

10. MySpace

11. Quora

12. Sanctioned Suicide

13. Forums

14. Blogs

15. Not specified

16. Can’t remember / unsure

17. The Mix

18. UseNet

19. Bodies Under Siege

20. WhatsApp

21. Health Unlocked

22. Childline

23. Piczo

24. LiveJournal

25. NSHN website/forum

26. Netflix

27. Sony Sketch

28. Online news sites

29. Pro-Ana websites

30. Star Doll

31. Snapchat

32. Side-by-Side

33. Pro-suicide website

34. Telegram

35. Information and help sites

36. Social Anxiety Animo

37. Gore sites

38. DeviantArt

39. Online support groups

40. 4Chan

41. Ruinyourlife.org

42. Suicide forums

43. Self-harm website

44. Other

45. Not relevant

Type of content:

| 10. Visual user-generated content | 101. Images/videos of someone after they have died by suicide |
| --- | --- |
|  | 102. Images/videos of someone taking their own life |
|  | 103. Images/videos of suicide methods |
|  | 104. Memes about suicide |
|  | 105. Online polls |
|  | 106. Videos of ‘interventions’ recorded by bystanders |
|  | 107. Other visual user-generated content |
| 20. Descriptive user-generated content | 201. Sharing of active plans to die by suicide, which may or may not include detailing the method |
|  | 202. Sharing what has helped the person/coping strategies |
|  | 203. Talking about methods |
|  | 204. Describing being in the middle of a suicide attempt |
|  | 205. Describing being in a crisis moment |
|  | 206. Sharing traumatic/adverse experiences, and the relationship between these and the person’s suicidality |
|  | 207. Describing having just attempted to take one’s own life – reaching out for someone to talk to |
|  | 208. ‘Partner’ threads |
|  | 209. Sharing (drafts of) suicide notes |
|  | 210. Goodbye threads |
|  | 211. Countdowns |
|  | 212. Sharing hospital experiences related to suicide |
|  | 213. Sharing stories about an offline ‘intervention’ |
|  | 214. Experiences and opinions about campaigns/services |
|  | 215. Suicide challenges |
|  | 216. Videos of people who have previously attempted to take their own lives, survive, and are talking about the impact this had on them |
|  | 217. Videos/vlogs/blog posts/descriptive posts about recovery/hope messages |
|  | 218. Pro-choice vs. pro-life discussions |
|  | 219. Discussing reasons for joining the online community/seeking online content |
|  | 220. Discussing other communities’ online suicide content |
|  | 221. Talking about the difficulties of/seeking information on supporting someone who is suicidal |
|  | 223. Discussions from friends/relatives about being bereaved by suicide |
|  | 224. Jokes about suicide or suicidal ideation |
|  | 225. Experiences of suicidal feelings and attempts |
|  | 226. Other descriptive user-generated content |
| 30. News articles and other media | 301. Media/academic discussions theorising a rise in the suicide rate |
|  | 302. Media discussions of harmful content on social media |
|  | 303. News story about a suicide – may or may not include detail of method |
|  | 304. Other news or media content |
| 40. Content produced by organisations | 401. Campaigns relating to suicide or mental health |
|  | 402. Intervention banners/resources, such as produced by Samaritans or platforms themselves |
|  | 403. Campaigns about or petitions to remove ‘harmful’ social media content |
|  | 404. Government or policy announcements relating to suicide and social media |
|  | 405. Announcements about a suicide e.g., rail companies tweeting |
|  | 406. Other types of content produced by organisations |
|  | 407. Entertainment / streaming content |
| 50. Can’t remember |  |
| 60. Don’t want to say |  |
| 70. Not specified |  |
| 80. Not relevant |  |
| 90. Self-Harm content | 901. Experiences of self-harm or desire to self-harm |
|  | 902. Descriptions and/or visual self-harm content |
|  | 903. Other self-harm content |
| 100. Other (specific descriptions) | 1001. Specific type, but unclear / unknown general type |
|  | 1002. Specific description but does not fit other general type |
|  | 1003. Specifically methods or locations, but unclear general type |
| 200. Audio content | 2001. Music compilation |
|  |  |
|  |  |

Context:

1. Graphic

2. Romanticised

3. Distressed

4. Depressed

5. Trivialising [Usually in the context of memes]

6. Expressing/documenting feelings or experiences [Unsure whether we need this one]

7. Detached or instructive (e.g., methods information)

8. Eating disorder related

9. Help-seeking

10. Recovery-focused [for lived experience accounts of recovery, celebrating recovery, etc]

11. Encouraging suicide

12. Supportive

13. Informative/reporting/educational (e.g., research or news)

14. Other

15. Not specified/unclear

16. Suicidal

Q72 When you FIRST saw suicide content online, how did it make you feel? Please tick as many as apply: Other – please specify

1. Desire to help

2. Numb or emotionless

3. Worried/scared/concerned

4. Mixed emotions

5. Empathy (inc. Sadness for others)

6. Didn’t feel anything/not bothered/indifferent

7. Angry

8. Triggered

9. Depressed/sad

10. Entertained (e.g., laughed)

11. Sickened or revolted

12. Unsure

13. Other (e.g., trendy)

14. Can’t remember

15. Not relevant

Q78 Usually, how does seeing online content about suicide make you feel now? Please tick as many as apply: Other – please specify

1. Desire to help

2. Numb or emotionless

3. Worried/scared/concerned

4. Mixed emotions

5. Empathy

6. Didn’t feel anything/not bothered/indifferent

7. Angry

8. Triggered

9. Depressed or sad

10. Entertained

11. Sickened or revolted

12. Curious

13. More informed

14. Vulnerable

15. Dependent on content

16. Reflective or proud of self

17. Validated

18. Jealousy

19. Unsure

20. Other

21. Can’t remember

22. Not relevant
